# Machine learning-driven critical care decision making

**DOI:** 10.1177/01410768221089018

**Published:** 2022-04-07

**Authors:** James T Coates, Christiaan de Koning

**Affiliations:** 1 Harvard Medical School & Massachusetts General Hospital, Boston, MA, 02114, USA; 2 Saïd Business School, University of Oxford, Oxford, OX1 1HP, UK

Artificial intelligence (AI)-based technologies for healthcare are being developed increasingly day-by-day.^1^ While they are often used interchangeably, machine learning (ML) encompasses a subset of techniques within the broader field of AI often being more complex and having more abstruse physical interpretations (Figure 1).^2^ An increasingly recognised necessity for the robust and successful implementation of ML-based platforms in healthcare is relevant, high-quality data around which such platforms can be trained, tested and *trusted* (Figure 2(a) and (b)). Centralised healthcare systems such as the United Kingdom’s National Health Service (NHS) are therefore uniquely positioned to exploit the advantages of ML-based tools given their population-wide accessibility, standardisations of data collection and regulatory governance structures. Application of ML to solve and optimise challenges in systems such as the NHS is as far from a mature concept as it is novel; however, the systemic and methodological impact of the use of such technologies in critical care decision making, as well as pursuant legal ramifications, is only now coming to light and is worryingly opaque.

**Figure 1.**
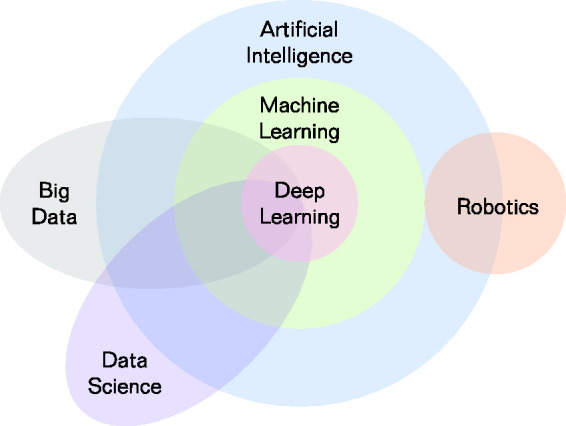
Overview of major disciplines within and related to artificial intelligence. Adapted from literature.^3^.

**Figure 2.**
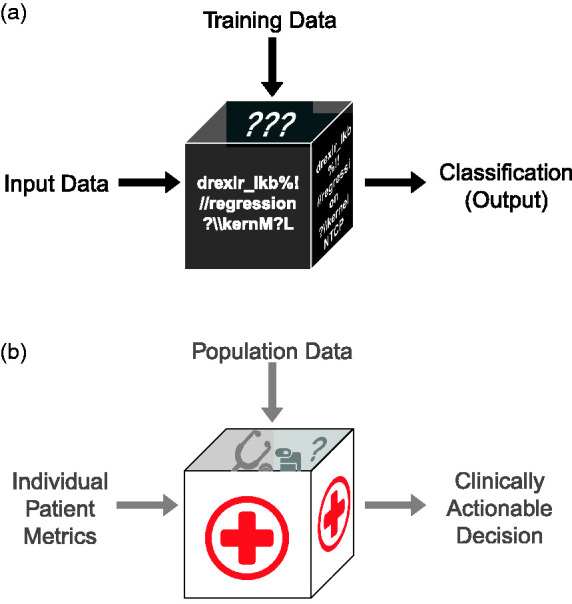
Projection of the machine learning black box algorithm onto clinical practice. (a) Predictive models are *trained* on datasets and then individual input data *tested*, yielding a classification (*output*). (b) In the case of clinical medicine, individual patient metrics can be *tested* after constructing models on larger datasets, yielding a clinically actionable decision but doing so with limited mechanistic transparency or explainability.

Future policy choices relating to ML-driven healthcare technologies will be predicated on those made today as it is unarguably a period of genesis for the field.^4^ Shortcomings in governance, legislation, ethical codes, liability structures and policies relating to implementation risk deterring private enterprises from engaging. This also renders the field less attractive to potential innovators and investors.^5^ Regulatory shortcomings furthermore leave national infrastructure open to abuse by for-profit entities. ML-driven healthcare technological platforms, for example, may seek to optimise measures such as cost or suppliers. These are readily exploitable if the decisions rely upon relationships that cannot readily be deconvoluted or rationally understood by decision makers (*trusted*). However, in the case of critical care medicine, decisions are less likely to be as reversible and innocuous.^6^ Rather, they are associated with acute consequences. This renders the role for critical care technologies that use ML-based algorithmic decision making ambiguous, at best.

In critical care, ML-based platforms may not be readily understood by medical practitioners.^7^ This is undoubtedly the case for stakeholders lacking knowledge of the underlying nature of AI but applies strongly to ML-based techniques that lack intelligibility due to their inherent ‘black box’ nature.^8,9^ Consequently, the use of ML-driven platforms for decision making, rather than only decision support, seriously risks limiting, or even corrupting, knowledge transfer from physician to patient, which otherwise normally underpins informed decision making in Western medicine.^10,11^ A solution may be to introduce bioinformaticians into the decision-making framework as professionals that can readily translate to administrators, practitioners and patients the limits and readouts of machines.

The current decision-making framework of the NHS can be aggregated into one of three overarching categories: clinical, patient-led or shared (Figure 3(a)).^12^ In the case of clinical decision making, decisions rest on the expertise, knowledge, experience and guidance of healthcare practitioners building on research, experience and data. This may involve consultation with multiple practitioners, especially in complex cases, and may be policy-driven for socioeconomic, ethical or legal reasons. Patient-led decision making is often used when options are available for a patient with no absolute indications of one over another. The patient is presented multiple treatment options and then given the risks and benefits of each as per the interpretation of the physician of the associated data. Shared decision making on the other hand ensures that individuals are supported to make decisions that are right for them through a collaborative process with medical practitioners.

**Figure 3.**
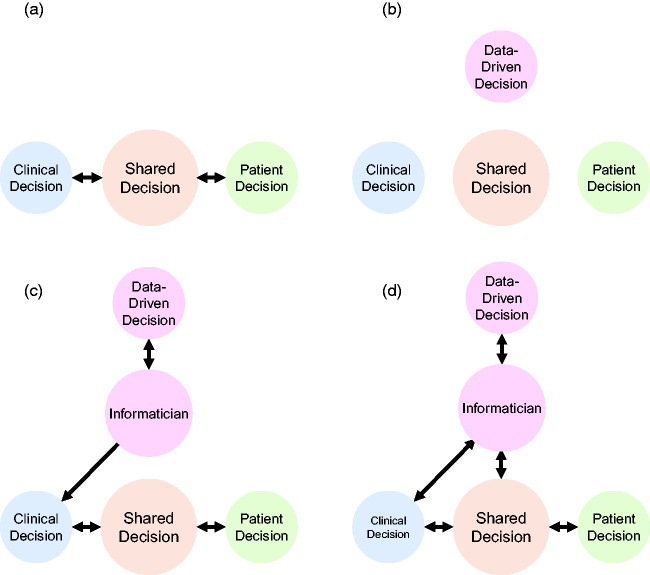
Current and proposed shared decision-making framework for the NHS. (a) Current shared decision-making framework whereby decisions can be made my clinical professionals, by patients or mutually. (b) Anticipated relative importance of data-driven decision making compared to clinical and patient-only directed decisions. (c) Proposed workflow for integration of informaticians into the shared decision-making framework (data-driven, clinician-led). (d) Proposed role for informaticians in shared decision making, which does not preclude some preference of data-driven decisions in shared decision making regardless of intelligibility.

In order to facilitate the introduction of ML-based technologies, we propose a fourth category of decision making: *data-driven* (Figure 3(b)). Data-driven decision making can broadly be considered to include the use of AI but especially ML-based technologies that make decisions devoid of most patient and clinical inputs and are not easily interpretable. Importantly, in the case of shared decision making, ML-based technologies could be incorporated via bioinformaticians that participate in the shared decision-making process via consultation with clinical practitioners (Figure 3(c)). Eventually, decisions and data could be returned to the ML platforms through the bioinformaticians for further refinement or model adjustment (real-life *back propagation*) thereby continually ‘learning’ and improving performance without compromising clinician-led decision making or liability (Figure 3(d)).

Healthcare workflow changes such as the ones proposed herein are only a few of the possible solutions to the conundrum posed by ML-driven decision making for critical care. It is realistic that any major change to decision making frameworks may give rise to friction and resistance such that lock-in of the current system can be expected for some time. Practitioners and patients are familiar with current practices but might also be invested in them for a variety of reasons.^12^ To shift away from established practices, trust of stakeholders has to be gained. In the case of the NHS, the use of informaticians could bridge the gaps in policy and liability left by the adoption of complex ML-based technologies while simultaneously providing avenues to promote trust. In this sense, the inclusion of informaticians into critical care decision- making frameworks is not dissimilar to the use of human drivers for on-road testing of autonomous vehicles – humans can record performance metrics as a vehicle is in action, yes, but they also create trust in society and facilitate responding to emergencies, errors, accidents and other inevitable, unforeseeable incidents.

## Declarations

### Guarantor

JC.
